# Country-Wide Surveillance of Molecular Markers of Antimalarial Drug Resistance in Senegal by Use of Positive Malaria Rapid Diagnostic Tests

**DOI:** 10.4269/ajtmh.17-0021

**Published:** 2017-08-28

**Authors:** Magatte Ndiaye, Doudou Sow, Sidsel Nag, Khadime Sylla, Roger Clement Tine, Jean Louis Ndiaye, Aminata Collé Lo, Oumar Gaye, Babacar Faye, Michael Alifrangis

**Affiliations:** 1 Service de Parasitologie–Mycologie, Faculté de Médecine, Université Cheikh Anta DIOP, Dakar, Sénégal;; 2 Centre for Medical Parasitology, Department of Immunology and Microbiology, University of Copenhagen, Copenhagen, Denmark;; 3 Department of Infectious Disease, Copenhagen University Hospital, Copenhagen, Denmark

## Abstract

In Senegal, antimalarial drugs used in treatment and prevention of malaria are one of the main reasons for the current success in controlling malaria. However, the successful control of malaria is highly dependent on continued effectiveness of these drugs which may be compromised by the spread of drug resistance. Therefore, surveillance of drug resistance in the malaria parasites is essential. The objective of this pilot study was to test the feasibility of routinely sampled malaria rapid diagnostic tests (RDTs) at a national scale to assess the temporal changes in the molecular profiles of antimalarial drug resistance markers of *Plasmodium falciparum* parasites. Overall, 9,549 positive malaria RDTs were collected from 14 health facilities across the country. A limited random set of RDTs were analyzed regarding *Pfcrt* gene polymorphisms at codon 72–76. Overall, a high but varied prevalence (> 50%) of the wild-type CVMNK haplotype was observed including a higher CVMNK prevalence in the northern part (75%) compared with the southern part of the country (59%). With caution, the study provides a proof of concept that reuse of discarded *P. falciparum* positive RDTs can be applied in large-scale surveillance of antimalarial drug resistance.

In Senegal, chloroquine (CQ) was the first-line treatment for uncomplicated malaria until 1998. High rates of CQ treatment failures and an increased risk of childhood malaria deaths^1^ prompted Senegalese health authorities to abandon CQ in 2003. Sulfadoxine–pyrimethamine (SP) plus amodiaquine (AQ) was then introduced as the first-line treatment for uncomplicated *Plasmodium falciparum* malaria despite the fact that low numbers of SP and AQ treatment failures were documented in the country when used alone.^2–4^ However, because of resistance against widely used antimalarial drugs, most countries have been forced to introduce artemisinin combination therapies (ACTs) for malaria treatment. Following World Health Organization (WHO) recommendation, in 2006, the Senegalese health authorities changed to fixed dose combinations of ACTs; artemether–lumefantrine and artesunate plus AQ (depending on availability) for malaria treatment. In all sub-Saharan African (SSA) countries with endemic *P. falciparum* malaria, ACTs have now been implemented and are highly efficacious and, until now, without showing any major signs of emerging ACT resistance. Despite the decrease in malaria burden in SSA, mainly as a result of successful control interventions such as large-scale distribution of insecticide-treated bed nets and treatment with ACTs, it is becoming crucial to identify emerging drug resistance in an attempt to delay ACT resistance from spreading to SSA from Southeast Asia where it is evident. Molecular surveillance of drug resistance in *P. falciparum* populations could in that aspect become essential because this could provide an early warning sign of emergence of ACT resistance before clinical treatment failures are observed. Geographical mapping of molecular surveillance data can furthermore detect temporal changes of resistance, for instance, increased susceptibility to drugs that have been abandoned due to drug resistance, such as CQ.

In many malaria-endemic areas, less than half of patients with suspected malaria infections are truly infected with a malaria parasite.^5^ Therefore, parasitological confirmation by microscopy or rapid diagnostic tests (RDTs) of all suspected malaria cases is recommended by WHO before antimalarial treatment for malaria positives are begun.^6,7^ Accordingly, from September 2007, through the National Malaria Control Program (NMCP), Senegal has implemented the use of RDTs into the revised national policy for management of febrile illnesses.^8^ Furthermore, this has the advantage of restricting ACT use to confirmed malaria cases only, resulting in reduced ACT drug pressure and thus limiting the risks of drug resistance development and spread.^9^

Previous studies on molecular markers of drug resistance have largely relied on extraction of DNA from blood of malaria-infected subjects sampled on filter papers. Such samples have been obtained through malaria epidemiological or clinical trial studies whereby the filter papers are sampled as an integrated activity of these trials. However, because of declining malaria in SSA, it has become increasingly difficult to obtain parasite-positive samples. Furthermore, the sampling and resulting molecular data have depended on where these trials have been executed and have resulted in geographically sporadic distribution with some regions/sites contributing to significant molecular knowledge whereas others are not represented at all. Because studies have shown that it is possible to obtain molecular data using RDTs,^10,11^ instead of relying on trials for sampling of blood for molecular surveillance, routine sampling, and “recycling” of the positive malaria RDTs at sentinel sites could be an attractive alternative. Such an approach is most likely highly cost effective and would potentially create a repository for molecular surveillance. Such setup would additionally create a sampling and environmentally safe disposal of used RDTs that otherwise would be thrown away.

The aim of this study was to pilot test a routine sampling procedure for obtaining used RDTs from sentinel sites across Senegal and measure the prevalence of single nucleotide polymorphisms of one marker of antimalarial drug resistance, the *Pfcrt* gene related to CQ resistance in a subset of samples.

This study was conducted in 14 health facilities across Senegal, namely from the southern zone: Saraya, Kédougou, Velingara, and Tambacounda; from the central zone: Kaolack and Ndoffane; from the western zone: Dakar (including Guédiaway and Pikine); and from the northern zone: Richard Toll, Podor, and Louga (including Sakal health center) (see Figure 1). All health posts within each district were included in the study. Before RDT collection, information meetings with health authorities, including nurses and community health workers and the NMCP, were held to explain the objectives and importance of the study. From July 2013 through 2014, malaria positive RDTs (SD bioline) were collected, and site, district, gender, age, and month/year of sampling were noted on each RDT. The collected RDTs were stored into boxes containing silica gel and shipped monthly to the central laboratory at the Parasitology Department in the University of Cheikh Anta Diop in Dakar.

**Figure 1.**
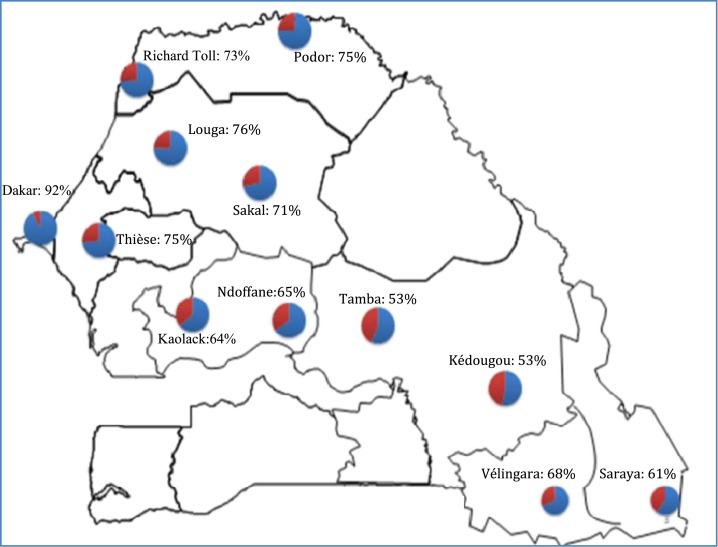
Prevalence of *Pfcrt* haplotypes (CVMNK) across Senegal. Blue: *Pfcrt* wild-type (CVMNK) haplotype also noted as actual percentage. Red: *Pfcrt* mutant (CVIET) haplotype. This figure appears in color at www.ajtmh.org.

Among these, a limited set of positive RDTs were randomly selected from each health facility and analyzed further regarding polymorphisms at codon 72–76 of the *Pfcrt* gene. The RDTs cassettes were opened using sterile scissors and forceps, and the nitrocellulose strips taken out. The strips were cut into two pieces, and the portions containing the sample-blotting site were used for DNA extraction and placed in a 96 deep-well plate following guidelines.^12^ In between each sample, the forceps and scissors were washed in ethanol, burned, and dried on a clean tissue paper to minimize cross-contamination during sample preparation. DNA extraction was performed on the portion of strips containing blood using the Chelex-100 method as previously described.^13^ For *Pfcrt* genotyping at codons 72–76 (only identifying the CVMNK wild-type and CVIET mutant haplotypes), a nested polymerase chain reaction (PCR), followed by a sequence-specific oligonucleotide probe (SSOP)-enzyme-linked immunosorbent assay (ELISA), was applied as described.^14^

Overall, 9,549 (6,000 in 2013 and 3,549 in 2014) positive malaria RDTs were collected from the health facilities across the country as shown in Table 1. Some health facilities provided more malaria positive RDTs than other districts, to some extent reflecting differences in levels of transmission between the districts. Combining the data, malaria, based on the positive RDTs collected, was more prevalent in the southern zone (*N* = 4,522) compared with the central (*N* = 2,947), western (*N* = 1,372), and northern zones (*N* = 708).

**Table 1:** RDT collected and analyzed and PCR efficacy

Area (zone)	Districts	Positive RDTs collected	RDTs analyzed	*Plasmodium falciparum* positive by PCR
Southern	Kédougou, Vélingara, and Tambacounda	4,522	176	168
	Saraya			
Central	Kaolack	2,947	132	126
	Ndoffane			
Western	Dakar (Guédiaway Pikine)	1,372	88	82
	Thiése			
Northern	Richard Toll	708	176	162
	Podor			
	Sakal			
	Louga			

RDTs = malaria rapid diagnostic tests; PCR = polymerase chain reaction; *Plasmodium falciparum* positive by PCR: samples positive by PCR and SSOP-ELISA where a haplotype of the *Pfcrt* gene at codon 72–76 could be determined.

In total, the haplotype at codons 72–76 of the *Pfcrt* gene using the PCR-SSOP-ELISA was successfully determined in 94.7% (538/572) of samples as shown in Table 1. Overall, a high but varied prevalence (> 50%) of the wild-type CVMNK haplotype was observed in all examined health facilities. There was a clear tendency for higher prevalence of the CVMNK haplotypes in the northern and northwestern part of the country (for instance, Richard Toll: 74%; Podor: 78%; Louga: 76%, and Dakar: 92%) compared with the southern part (Tambacounda: 53% and Kédougou: 61%) though, Vélingara was at 68% (see Figure 1).

Increasing prevalence of CQ sensitive *P. falciparum* populations has been shown before in Senegal,^15^ and the data presented here complement these findings, and eventually, CQ may be attempted to be reintroduced again in combination with another drug and, for instance, only be given to certain patient groups. However, the wide differences observed across the country underscores the validity (and necessity) of continued monitoring of molecular markers if the markers shall become operational and support drug policy.

In this study, we provided a proof of concept that reuse of discarded *P. falciparum* positive RDTs can be applied in large-scale surveillance of antimalarial drug resistance. Thus, sampling of these positive RDTs could provide a sample set in a magnitude and geographical distribution necessary to follow the development of drug resistance at a country or even on a multicountry level. Such data may eventually support drug policy authorities in decisions regarding choice of drugs and possibly limit the time between when a change in policy is necessary and when such a change actually is implemented. Furthermore, the RDT sampling procedure is as well a much cheaper approach than establishing procedures based on designated sampling of filter papers as these RDTs are already available. Lastly, the alternative reuse of RDTs furthermore deals with the increasing waste problem arising from the increasing use of RDTs.

However, there are some points of caution before RDTs sampling, and molecular analyses can be established as a routine procedure to provide essential data on antimalarial drug resistance. Despite that we were able to successfully genotype 94.7% of the samples, further studies using a larger batch of positive RDTs in subsequent experiments have not been as successful and less than 50% of the positive RDTs were PCR positive (data not shown). One reason for this is that the sampling and storage procedure (storage at room temperature with silica gel until use) could have hampered effective DNA extraction, and only RDT positives with high parasitemia (and thus large quantity of *P. falciparum* DNA) resulted in PCR positives. Possibly, the longer time the RDTs are stored (at high room temperatures) the lower is the chance to obtain successful PCRs. Preliminary data do suggest that long-term storage can decrease sensitivity of the RDTs for molecular analyses.

If this obstacle is solved, the molecular data should eventually provide crucial information that will aid drug policy makers. However, the data have to be interpreted and available soon after sampling which is usually not the case. For instance, between sampling and publication of molecular data on antimalarial drug resistance others have shown typically that there is a lag phase of 5 years.^16^ Thus, rapid assessment and provision of data (and not necessary awaiting scientific publication) are necessary if the molecular data should become operational. We used the PCR-SSOP-ELISA to obtain data, and although simple and relatively high-throughput, alternative methodologies using, for instance, next-generation sequencing platforms would, in particular, be important to implement as much focus should be on sequencing the *K13* gene associated with ACT resistance.^17,18^

Lastly, as no guidelines exist on how often surveillance surveys should be undertaken and what coverage of sentinel sites is sufficient to provide operational molecular data to guide antimalarial drug policy makers, this needs to be explored.
